# Posterior Horn Meniscus Centroid Position Is Altered Soon After Noncontact ACL Injury in Males and Females

**DOI:** 10.1002/jor.70208

**Published:** 2026-04-27

**Authors:** Benjamin T. Hamilton, Daniel Sturnick, Erin C. Argentieri, Niccolo Fiorentino, Timothy W. Tourville, Matthew Failla, Mack Gardner‐Morse, Pamela M. Vacek, Bruce D. Beynnon

**Affiliations:** ^1^ Department of Orthopaedics and Rehabilitation, Robert Larner M.D. College of Medicine University of Vermont Burlington Vermont USA; ^2^ Stryker Spine, Stryker Leesburg Virginia USA; ^3^ Department of Radiology and Imaging Hospital for Special Surgery New York New York USA; ^4^ Department of Mechanical Engineering University of Vermont Burlington Vermont USA; ^5^ Department of Rehabilitation and Movement Science University of Vermont Burlington Vermont USA; ^6^ Biomedical Statistics Research Core, Robert Larner M.D. College of Medicine University of Vermont Burlington Vermont USA

**Keywords:** ACL, meniscus, posterior horn, post‐traumatic osteoarthritis

## Abstract

Anterior cruciate ligament (ACL) injury and subsequent changes in the magnitude and distribution of contact stress about the articular surfaces of the knee are associated with post‐traumatic osteoarthritis. Soon after ACL injury, changes in tibial articular cartilage thickness occur that can be explained, in part, by abnormal positioning of the tibia relative to the femur. However, little is known about the effects of ACL injury on the positions of the menisci. The purpose of this case‐control study was to determine the effect of ACL injury on posterior horn meniscus centroid (PHMC) position relative to the tibia in males and females. ACL‐injured and matched control subjects with normal knees underwent bilateral magnetic resonance imaging soon after the index injury and prior to ACL reconstruction. The PHMC position was defined in three dimensions at the point of maximal tibial articular cartilage concavity in the lateral and medial compartments of the knee. In control subjects, there were no significant differences in PHMC position between knees in the lateral and medial compartments. In ACL‐injured knees, there were significant posterior‐directed changes in PHMC position in both compartments for males and females when compared to their contralateral normal knees. These changes in PHMC position may alter the distribution of articular cartilage contact stress and explain a portion of the changes in knee biomechanics and cartilage thickness that occur following ACL injury. In addition, these findings suggest that the menisci should not be used as landmarks to establish regions of interest when measuring cartilage thickness and matrix components.

## 1 Introduction

The contributions of anterior cruciate ligament (ACL) injury to the development of abnormal knee biomechanics [1, 2, 3] and post‐traumatic osteoarthritis (PTOA) are well‐documented [4, 5, 6]. ACL trauma is immediately debilitating and poses a threat to patients' future quality of life. With the highest incidence of severe knee trauma, including combined ACL and meniscal injuries, occurring in individuals between the ages of 15 and 25 years [7, 8], many young, active individuals are at an increased risk of developing symptomatic PTOA within 15 years of the index trauma [9]. ACL injuries frequently include concomitant meniscus trauma. While decreased height of the lateral posterior meniscal horn [10], as well as reduced posterior horn meniscus wedge angle, are associated with increased risk of suffering a first‐time, noncontact ACL injury [11], little is known about the effects of ACL injury on meniscal position relative to the tibiofemoral joint in vivo. Likewise, little is known about the effect of the position of the menisci relative to the tibiofemoral joint on the underlying mechanisms of PTOA onset and progression.

The menisci are integral fibrocartilage structures that attenuate the transmission of impulsive loads and distribute contact stress across the tibiofemoral joint [12, 13, 14]. The position of the menisci relative to the tibia and femur therefore influences how these intersegmental compressive and shear stresses are transferred across the tibiofemoral joint. To date, changes in contact biomechanics between the tibial and femoral articular surfaces following ACL injury have primarily been attributed to increases in anterior translation and internal rotation of the tibia relative to the femur during activity [2, 3, 15, 16]. These altered contact mechanics likely modify changes in articular cartilage thickness [17] and the balance between synthesis and cleavage of underlying matrix components [18] associated with the development of PTOA.

Our prior work using magnetic resonance imaging (MRI)‐based measurements of articular cartilage thickness revealed that both local thickening and thinning of the tibial articular cartilage occur very soon after ACL disruption [17]. Further, 4 years following ACL reconstruction (ACLR) with a bone‐patella tendon‐bone autograft, the area and magnitude of the changes (thickening and thinning) of articular cartilage about these same cartilage regions were found to increase substantially [19]. In both reports, a portion of the changes in articular cartilage thickness were associated with anterior‐directed positioning of the tibia relative to the femur in the ACL‐injured and reconstructed knees compared to the contralateral normal side at the time of MRI acquisition. However, it remains unclear how the position of the menisci relative to the tibia after the index ACL trauma modifies tibiofemoral contact biomechanics and produces changes in articular cartilage thickness associated with PTOA pathogenesis. This served as the motivation for our current study, which was to characterize the position of the menisci relative to the tibia soon after ACL injury and prior to ACLR. Gaining an improved understanding of the factors that contribute to the development and early progression of PTOA holds the most promise for informing the development of prevention and treatment strategies for this disease.

The purpose of this study was to assess the effect of noncontact ACL injury on posterior horn meniscus centroid (PHMC) position in the lateral and medial compartments of the tibia. This was done by determining if there are differences in PHMC position between the injured and uninjured knees of ACL‐injured subjects that are larger than the corresponding differences between the knees of matched control subjects with normal knees and no history of injury or disease. We hypothesized that increased posterior‐directed PHMC position relative to the tibia would be observed in ACL‐deficient knees compared to contralateral normal knees in males and females, and that there would be no comparable side‐to‐side differences in PHMC position between the corresponding normal knees of matched control subjects.

## 2 Methods

This investigation was approved by the institutional review board at the University of Vermont, and all study participants and/or their legal guardians provided written informed consent prior to data collection. This study is based on additional analysis of MRI data originally collected in a prospective cohort study that used a nested, matched case‐control sampling [10, 20, 21, 22, 23]. The MRI data obtained in this prior investigation were used to study the effect of the index ACL trauma on knee articular cartilage thickness and to develop a multivariate model of the knee geometry risk factors associated with noncontact ACL injury [11, 17, 24].

The recruitment, entry criteria, study design, protocol, and participant demographic information have been described [20]. In brief, the athletic trainers who worked with sports teams at 28 high schools and 8 colleges in our region prospectively followed athletes and approached potential subjects about participating in this study when a noncontact ACL injury was suspected. An injury was classified as noncontact if it did not involve direct impact to the knee. At the high school level, the following sports were studied: soccer, basketball, and lacrosse for boys and girls, and football and field hockey for boys and girls, respectively. At the college level, men's and women's rugby, as well as women's volleyball, were studied in addition to the previously mentioned high school sports.

Subjects with suspected noncontact ACL injuries that occurred during participation in an organized sport were recruited for the study after follow‐up with an orthopedic surgeon who made a clinical diagnosis of a complete ACL disruption that was confirmed with arthroscopic visualization at the time of ACLR. ACL‐injured subjects with a history of ACL injury or other severe trauma (bone, ligament, meniscus, or articular cartilage) to either knee were excluded. At the time an ACL injury was confirmed, control subjects with normal knees and no history of injury or disease were selected from each injured subject's team to enable one‐to‐one matching based on sport, level of play, and subject sex. This approach was used to control for exposure to activity and intrinsic/extrinsic risk factors for ACL injury between the ACL‐injured and control subjects. All control subjects had normal knees with no history of trauma or disease. In addition, they had normal menisci and articular cartilage that was confirmed by MRI examination.

Bilateral MRI scans were acquired from 88 first‐time ACL‐injured subjects (27 male and 61 female) soon after the index trauma, but before ACLR, and from the 88 matched uninjured controls (27 male and 61 female) with the approach that has been described [10, 11, 20]. Briefly, the scanning volume was aligned with anatomical axes of the knee, and sagittal T1‐weighted fast field echo (FFE) scans were acquired three‐dimensionally with a voxel size of 0.3 mm × 0.3 mm × 1.2 mm. All MRIs were acquired while subjects were in a supine, non‐weight‐bearing position with their knee in extension using the same Phillips Achievea 3.0 T MRI system (Phillips Medical Systems) and the same 8‐channel SENSE knee coil. Tibial plateau subchondral bone, tibial articular cartilage surface, and posterior horn meniscus boundaries were then segmented manually using a Cintiq 21UX digitizing tablet (Wacom Technology Corporation, Vancouver, WA, USA) with OsiriX software (Pixmeo, Geneva, Switzerland, version 5.5.1) [17].

**Figure 1:**
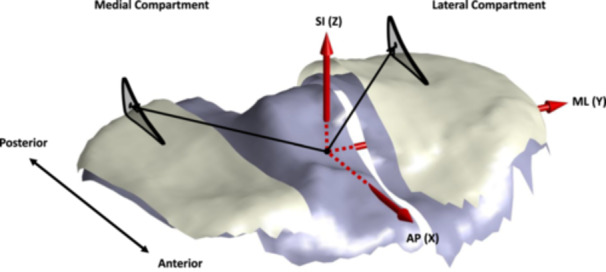
Tibia sagittal‐plane digitized bone (blue) and cartilage (white) surfaces within the pre‐established bone‐fixed tibial coordinate system. The anterior‐posterior (AP), medial‐lateral (ML), and superior‐inferior (SI) axes of the coordinate system are shown in red. Lateral and medial meniscus slices (outlined in black) and corresponding posterior horn meniscus centroids (PHMCs) (black dots) are shown mid‐compartment for the MRI slice of maximal tibial articular cartilage concavity. The location of each PHMC was determined in three dimensions within the tibial coordinate system of each knee. The distance from the origin of the tibial coordinate system to the lateral and medial PHMCs (black arrows) is the resultant vector of the AP (*X*), ML (*Y*), and SI (*Z*) components of each PHMC location in three dimensions. The tibial coordinate system used has been described previously [21].

**Figure 2:**
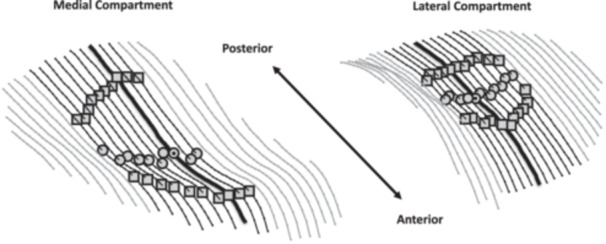
Determination of MRI slice at the point of maximal tibial articular cartilage surface concavity in the lateral and medial knee compartments. The anterior and posterior peaks of the tibial articular cartilage were defined as the most superior points along the anterior and posterior cartilage rims (squares) in the lateral and medial compartments. The local minima for each sagittal slice (circles) and the points of greatest articular cartilage concavity (dot within circle) are also shown in both compartments. The thickened black lines highlight the corresponding mid‐compartment slices on which the posterior meniscal horns were segmented to determine the lateral and medial posterior horn meniscus centroid (PHMC) positions for each knee.

**Figure 3:**
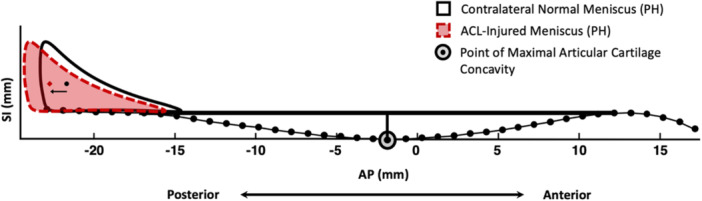
Difference in medial compartment posterior horn meniscus centroid (PHMC) position between ACL‐injured knees and matched contralateral normal knees. Sagittal profiles of the posterior horns (PH) of the segmented menisci at the MRI slice of greatest tibial articular cartilage surface concavity for the ACL‐injured knee (dotted red line) and contralateral normal knee (solid black line) are plotted in the AP and SI directions. The tibial articular cartilage surface is outlined by the dotted black line. The point of maximal articular cartilage concavity (dot within circle) was defined as the greatest perpendicular distance (vertical line) between the articular cartilage surface (black dots) and the line constructed tangent (horizontal line) to the anterior and posterior peaks of the tibial articular cartilage surface.

Custom MATLAB code was developed and used to post‐process the tibial subchondral bone, tibial articular cartilage surface, and posterior horn meniscus segmentations and transform these data from the coordinate system of the MRI scanner into the coordinate system of the tibia generated for each MRI acquisition using the approach we have described [21] (Figure 1, Figures S2 and S3). This enabled the meniscus segmentations to be localized within the tibial coordinate system of their corresponding knees and standardized the MRI slice location relative to the alignment of the tibia in the MRI scanner. PHMC measurements within the same knee, within subjects, and between case and control subjects could then be made in a reliable and reproducible manner. Using the approach we have described [10, 21], a single sagittal MRI slice at the point of maximal tibial articular cartilage concavity was identified in the lateral and medial compartments of the knee, respectively. PHMC position was then determined in three dimensions using the corresponding segmentation of the posterior meniscal horn from this MRI slice in each compartment (Figures 2 and 3). The data were processed to determine each PHMC position within the bone‐fixed tibial anatomic coordinate system using a standardized approach [17].

### 2.1 Statistical Analysis

For the PHMC position analyses, all knees were analyzed in the previously described bone‐based tibial coordinate system where the *x*‐axis was defined as the anterior‐posterior (AP) directed axis, the *y*‐axis defined as the medial‐lateral (ML) directed axis, and the vertical *z‐*axis defined as the superior‐inferior (SI) directed axis (Figure 1). Positive values designated anterior‐, lateral‐, and superior‐directed differences in PHMC position between knees, while negative values represented posterior‐, medial‐, and inferior‐directed differences in PHMC position between knees.

In ACL‐injured subjects, within‐subject side‐to‐side differences in PHMC position relative to the origin of the tibial coordinate system were calculated by subtracting the PHMC position of each contralateral normal knee from the PHMC position of its corresponding ACL‐injured knee in the AP, ML, and SI directions (i.e., ACL‐injured knee minus contralateral normal knee). Paired, two‐tailed *t*‐tests using a significance level of 0.050 were performed for the lateral and medial knee compartments of the male and female groups to determine if there were significant differences in PHMC position between ACL‐injured and contralateral normal knees (Figure S1). The same analyses were performed to determine and compare PHMC position differences between the corresponding normal knees of control subjects (Figure S1). In addition, differences in PHMC position within ACL‐injured cases and within uninjured controls were compared using a paired, two‐tailed *t*‐test since the cases were matched to the controls. In control subjects, each normal knee was matched by side corresponding to the sidedness of the ACL injury to enable consistency in the difference calculations. PHMC position differences between normal knees were calculated by subtracting the position of Normal Knee 2 (matched by the side to the contralateral normal knee of the case subject) from Normal Knee 1 (matched by the side to the ACL‐injured knee of the case subject) in the AP, ML, and SI directions (Figure S1). For example, if a case subject had an ACL injury to his or her left knee, the PHMC position of the right knee of the matched control subject (Normal Knee 2) would be subtracted from the PHMC position of the control subject's left knee (Normal Knee 1). This process was repeated in all three anatomic directions (AP, ML, and SI).

The relative positions of the tibial and femoral coordinate system origins from ACL‐injured and contralateral normal knees were also evaluated to determine what proportion of the change in PHMC position relative to the tibia soon after ACL injury could be attributed to the position of the tibia relative to the femur (Supplement 2, Tables S1 and S2, and Figures S2 and S3). Pearson coefficients (*r*) were calculated within ACL‐injured subjects to determine if there was a significant correlation between the position of the tibia relative to the femur (measured as the vector between the origins of the coordinate systems located in each bone) and PHMC position relative to the tibia (measured as the vector between the PHMC and origin of the tibial coordinate system) in the lateral and medial compartments.

## 3 Results

### 3.1 Lateral and Medial PHMC Positions Within ACL‐Injured Subjects: Comparison of Injured and Contralateral Normal Knees

In both males and females, the lateral PHMCs were located more posteriorly relative to the origin of the tibial coordinate system in the ACL‐injured knees than in their contralateral normal knees (Figures 1, 2, 3). Specifically, in the AP direction, the lateral PHMCs of ACL‐injured knees were located an average of 1.84 mm (*p* < 0.001) and 0.61 mm (*p* = 0.009) more posteriorly, with ranges of 10.2 and 9.5 mm for males and females, respectively (Table 1). Analysis of the lateral knee compartment data from male subjects also revealed a significant inferior‐directed shift in PHMC position of 0.60 mm (*p* = 0.046) in ACL‐injured knees compared to the contralateral normal side (Table 1). There was no significant difference in the SI PHMC position for female subjects. Similarly, there were no lateral compartment differences in ML PHMC position between ACL‐injured and contralateral normal knees for either males or females (Table 1).

**Table 1:** Mean differences (95% CI) in posterior horn meniscus centroid (PHMC) position between ACL‐injured and contralateral normal knees within case subjects for male and female groups.

		ACL‐injured subject side‐to‐side differences					
		Lateral compartment			Medial compartment		
		AP (*X*) (mm)	ML (*Y*) (mm)	SI (*Z*) (mm)	AP (*X*) (mm)	ML (*Y*) (mm)	SI (*Z*) (mm)
Male	Mean PHMC position (95% CI)	−1.84 ± 0.47	0.41 ± 0.89	−0.60 ± 0.31	−1.31 ± 0.52	0.33 ± 0.42	−0.17 ± 0.18
	Max	2.6	7.0	1.8	3.8	5.0	2.3
	Min	−7.6	−8.0	−5.0	−6.3	−3.0	−1.8
	SD	2.24	4.26	1.50	2.50	2.02	0.88
	*p*	< 0.001	0.624	0.046	0.012	0.399	0.323
Female	Mean PHMC position (95% CI)	−0.61 ± 0.37	0.54 ± 0.69	0.06 ± 0.26	−1.10 ± 0.47	−0.31 ± 0.45	−0.02 ± 0.24
	Max	3.3	6.0	4.4	4.5	4.0	2.2
	Min	−6.2	−5.0	−2.5	−7.2	−5.0	−3.6
	SD	1.76	3.29	1.24	2.24	2.14	1.14
	*p*	0.009	0.205	0.713	< 0.001	0.260	0.904

*Note:* Positive mean differences indicate anterior‐, lateral‐, and superior‐directed differences in PHMC position between ACL‐injured and contralateral normal knees within case subjects. Negative mean differences represent posterior‐, medial‐, and inferior‐directed differences in PHMC position between ACL‐injured and contralateral normal knees. All values are displayed in millimeters (mm) and were calculated by subtracting the PHMC position of the contralateral normal knee from the PHMC position of the ACL‐injured knee within case subjects.

The medial PHMCs were also located more posteriorly relative to the origin of the tibial coordinate system in the ACL‐injured knees compared to the contralateral normal side for both sexes. In the AP direction, the PHMCs were located an average of 1.31 mm (*p* = 0.012) and 1.10 mm (*p* < 0.001) more posteriorly, with ranges of 10.1 and 11.7 mm for males and females, respectively (Table 1). There were no significant medial compartment differences in ML or SI PHMC position between ACL‐injured and contralateral normal knees in the male or female groups (Table 1).

### 3.2 Lateral and Medial PHMC Positions Within Uninjured Control Subjects: Comparison of Normal Knees

In the lateral compartment of the knee, no side‐to‐side differences in AP or ML PHMC position were found in the male and female controls (Table 2; both *p* > 0.050). Likewise, there were no significant differences in SI PHMC position between knees in the female controls (Table 2). However, in the male controls, the lateral menisci of the knees corresponding to the ACL‐injured knees of the matched cases were located 0.47 mm superiorly relative to the knees corresponding to the contralateral normal knees of the matched cases (*p* = 0.034).

**Table 2:** Mean side‐to‐side differences (95% CI) in posterior horn meniscus centroid (PHMC) position between normal control knees for male and female groups.

		Control subject side‐to‐side differences					
		Lateral compartment			Medial compartment		
		AP (*X*) (mm)	ML (*Y*) (mm)	SI (*Z*) (mm)	AP (*X*) (mm)	ML (*Y*) (mm)	SI (*Z*) (mm)
Male	Mean PHMC position (95% CI)	0.46 ± 0.30	0.26 ± 0.61	0.47 ± 0.23	0.01 ± 0.46	−0.07 ± 0.46	0.29 ± 0.33
	Max	4.0	5.0	2.2	4.4	4.0	3.1
	Min	−2.9	−7.0	−1.6	−4.1	−5.0	−2.6
	SD	1.44	2.90	1.10	2.22	2.22	1.57
	*p*	0.107	0.647	0.034	0.985	0.864	0.353
Female	Mean PHMC position (95% CI)	−0.12 ± 0.35	0.18 ± 0.62	−0.10 ± 0.28	−0.39 ± 0.38	0.26 ± 0.51	0.00 ± 0.20
	Max	3.6	7.0	2.8	5.5	6.0	2.0
	Min	−4.2	−8.0	−3.6	−4.3	−4.0	−2.9
	SD	1.66	2.99	1.35	1.80	2.44	0.96
	*p*	0.571	0.639	0.572	0.097	0.404	0.975

*Note:* Positive mean differences indicate anterior‐, lateral‐, and superior‐directed differences in PHMC position between the normal knees within control subjects. Negative mean differences represent posterior‐, medial‐, and inferior‐directed differences in PHMC position between normal knees within control subjects. All values are displayed in millimeters (mm) and were calculated using the matching scheme described in Figure S1.

Analysis of the data acquired from the medial compartment of the knee revealed no side‐to‐side differences in AP, ML, and SI PHMC positions between knees for either the male or female controls (Table 2; all *p* > 0.050).

### 3.3 Differences in PHMC Position Between ACL‐Injured and Uninjured Control Subjects

In the lateral compartment of the knee, male PHMC position differences between the injured and contralateral normal knees of the ACL‐injured subjects were significantly larger than the differences between the corresponding normal knees of the male control subjects in the AP (*p* = 0.001) and SI (*p* = 0.004) directions. Conversely, in females, the between‐knee differences in PHMC position in the lateral compartment did not differ significantly between the ACL‐injured and matched control subjects in all three anatomic directions (all *p* > 0.050). When combining the lateral compartment data from the male and female groups, the between‐knee differences in PHMC position were greater in the AP direction (*p* < 0.001) in ACL‐injured subjects than in uninjured control subjects.

In the medial compartment, PHMC position differences were larger between the injured and contralateral normal knees of the ACL‐injured subjects than between the corresponding normal knees of control subjects in the AP direction in the male (*p* = 0.034), female (*p* = 0.037), and combined sex (*p* = 0.003) groups. There were no significant differences in the magnitude of the PHMC position differences between the injured and contralateral normal knees of the ACL‐injured subjects and the corresponding normal knees of matched control subjects in the ML or SI anatomic directions (all *p* > 0.050) in the medial compartment.

### 3.4 Comparison of Differences in PHMC Position Between Male and Female ACL‐Injured Subjects

In the lateral compartment of the knee, significant differences in the magnitudes of ACL‐injured to contralateral normal side differences in PHMC position were found between males and females in the AP (*p* = 0.006) and SI (*p* = 0.033) directions, suggesting that a sex effect was present along these axes (Table 1). There were no differences in PHMC position between males and females in the ML direction in the lateral compartment (*p* > 0.050).

In the medial compartment of the knee, no significant sex effect of PHMC position was identified in the AP, ML, or SI directions (all *p* > 0.050).

### 3.5 Comparison of Differences in PHMC Position Between Lateral and Medial Knee Compartments Within ACL‐Injured Knees

There were no significant differences in PHMC position between the lateral and medial compartments of the ACL‐injured knees in the AP, ML, and SI directions for either males or females (Table 1; all *p* > 0.050).

### 3.6 Relationship Between PHMC Position and the Position of the Tibia Relative to the Femur

Within ACL‐injured subjects, significant anterior‐directed differences in the position of the tibia relative to the femur between ACL‐injured and contralateral normal knees were found in the male (mean: 4.28 mm) and female (mean: 2.35 mm) analyses (both *p* < 0.001; Table S1). Within control subjects with normal knees, no significant differences in AP tibial position relative to the femur were identified in males (*p* = 0.895; Table S2). However, in females, there was an anterior‐directed difference in the AP position of the tibia relative to the femur (mean: 0.45 mm) within control subjects (*p* = 0.031; Table S2).

We observed significant correlations between AP tibiofemoral and AP tibial‐PHMC position in both the lateral (*p* = 0.002) and medial (*p* = 0.002) compartments in the female ACL‐injured subject group, but not in the male ACL‐injured subject group (both *p* > 0.050). In female subjects, Pearson coefficients of 0.385 (lateral menisci) and 0.391 (medial menisci) indicated positive correlations between tibiofemoral position and tibial‐PHMC position in the lateral and medial compartments of the knee.

## 4 Discussion

The findings from this study indicate that increased posterior‐directed PHMC position occurs in the lateral and medial compartments of ACL‐deficient knees relative to contralateral normal knees in males and females soon after the index knee trauma. Our results also show there are no comparable side‐to‐side differences in PHMC position between the uninjured normal knees of control subjects. These findings suggest that the differences in PHMC position observed between ACL‐deficient and contralateral normal knees within ACL‐injured case subjects are produced by the index ACL injury.

We also found that changes in the position of the menisci relative to the tibia occurring soon after ACL disruption are substantial and highly variable between ACL‐injured and contralateral normal knees. For example, male PHMC position differences between ACL‐injured and contralateral normal knees had ranges up to 10.2 mm (AP) and 15.0 mm (ML) in the lateral compartment and 10.1 mm (AP) and 8.0 mm (ML) in the medial compartment. Likewise, female PHMC position differences within ACL‐injured subjects had ranges of 9.5 mm (AP) and 11.0 mm (ML) in the lateral compartment and 11.7 mm (AP) and 9.0 mm (ML) in the medial compartment. Similar findings were observed when considering males and females as a combined group (Table S3). This identification of variability in meniscus position change soon after ACL injury is important for the development of future qualitative and quantitative MRI post‐processing algorithms that define regions of interest (ROIs) in cartilage, specifically those that use the boundaries of menisci to establish ROIs. Current consensus guidelines [25] and existing literature [26] use the menisci as fixed and reproducible landmarks to establish different ROIs in articular cartilage when measuring cartilage thickness and matrix components in the context of osteoarthritis and PTOA pathogenesis. These methods could introduce considerable bias and confounding into such studies due to the highly variable position of the meniscus relative to the articular surfaces of the knee identified in the current study. Consequently, using the boundaries of the menisci to define tibial and femoral cartilage ROIs in ACL‐deficient and ACL‐reconstructed knees may not be a reliable approach in studies examining qualitative and quantitative MRI measures of articular cartilage. An alternative approach would be to use bone‐referenced ROIs. Bone‐referenced ROIs would eliminate the effects of variable meniscus position changes produced by injury and disease on establishing ROIs in articular cartilage. In addition, the highly variable changes in the position of the menisci may become an important consideration in longitudinal studies that include measurement time points after ACL injury and before surgery, and then subsequent follow‐up of the ACL‐reconstructed knee over time. The findings from the current study suggest the menisci would be in very different locations relative to the articular surfaces of the knee at these different time points. These findings may also have important clinical implications regarding the planning and execution of meniscal transplant surgery in patients with an injured or reconstructed ACL. Overall, posterior‐directed changes in PHMC position align with the involvement of the posterior horns of the menisci in the majority of meniscus injuries associated with ACL injury, as reported by Slauterbeck et al. [27] and Adams et al. [28]. In a sample of 722 patients who underwent ACLR, 903 meniscus injury combinations were identified. Of these combinations, 76.85% involved the posterior horn regions of the meniscus exclusively, while only 13.18% of tears were isolated to the anterior meniscal horns. Future work should be completed to better understand the correlation between posterior horn meniscus injuries and posterior‐directed changes in PHMC position soon after the index trauma and following reconstruction and return to preinjury activities.

Importantly, in the study of ACL‐injured subjects reported by Argentieri et al., when AP and ML tibial position relative to the femur were used as covariates in the analyses of abnormal tibial cartilage thickness distribution soon after the index injury and prior to surgery, 46% of the variability in abnormal cartilage thickness was attributed to abnormal position of the tibia relative to the femur at the time of MRI acquisition [17]. This indicates that approximately half (54%) of the variability in abnormal tibial cartilage thickness changes was unexplained. It is possible that more pronounced posterior positioning of the menisci relative to the tibia at the time of MRI acquisition could alter the distribution of tibiofemoral contact stress and in turn produce abnormal changes in the tibial cartilage thickness. Alternatively, the direct effect of abnormally high compression and shear forces at the time of ACL injury or altered biomechanics during the time between the index trauma and MRI acquisition could contribute to changes in the meniscus and articular cartilage thickness. Given the significant differences in PHMC position between ACL‐injured and contralateral normal knees and the potential association of these differences with changes in articular cartilage thickness within the same cohort, future studies involving articular cartilage thickness and matrix component concentrations should consider including PHMC position as a covariate in the statistical analysis in addition to tibiofemoral position. This may provide insight into how the position of the meniscus and/or changes in articular cartilage thickness and the underlying subchondral bone created by the index trauma contribute to PTOA pathogenesis.

The similarity in AP PHMC position differences between the lateral and medial knee compartments for ACL‐injured males and females suggests there were no significant differences in tibiomeniscal rotation between ACL‐injured and contralateral normal knees when the joint was scanned in full extension. This conclusion was supported by the absence of significant differences in internal or external rotation of the tibia relative to the femur between ACL‐injured and contralateral normal knees in males and females (Table S1; all *p* > 0.050).

Little is known about what proportion of PHMC position differences between ACL‐deficient and contralateral normal knees can be explained by the position of the tibia relative to the femur in males and females at the time of MRI acquisition. The correlation analysis we performed suggested that 14.8% (lateral menisci) and 15.3% (medial menisci) of the difference in AP PHMC position between ACL‐deficient and contralateral normal knees can be attributed to the position of the tibia relative to the femur. Therefore, about 85% of the AP‐directed changes in PHMC position soon after ACL injury and before reconstruction in females are not associated with the position of the tibia relative to the femur and can be attributed to other causes. This suggests changes in the position of the menisci relative to the tibia could be attributed to other factors, such as changes in meniscal morphometry produced by compressive and shear stresses associated with the index knee trauma. Future research is needed to better understand the factors that contribute to changes in PHMC position soon after ACL injury, in addition to tibiofemoral position.

An important strength of this study is that the MRI data acquired in the coordinate system of the scanner were transformed into a bone‐based tibial coordinate system for each knee. These transformations were accomplished with high side‐to‐side symmetry within and between subjects and with very strong inter‐examiner reliability [10]. Intraclass correlation coefficients (ICCs) were calculated to evaluate the reliability of our manual tibial articular cartilage segmentations among examiners. ICCs for intra‐observer reliability ranged between 0.75 and 0.96, indicating that our measurements were completed with good to excellent repeatability [10]. Further, the prospective study design enabled participants to be identified and enrolled soon after their first‐time noncontact ACL injury (median days post‐injury: 15). Nested case‐control sampling was used to select control subjects from the same athletic team of each ACL‐injured subject to enable matching based on exposure to pre‐injury activity, sex, age, sport, and level of play. An additional strength of this work was that all PHMC positions were determined relative to the tibia origin using a three‐dimensional approach. This enabled the PHMC positions to be determined in the AP, ML, and SI anatomically‐based directions in a reliable and reproducible manner without segmenting the entire menisci from the root insertions to the lateral and medial peripheral joint capsule interfaces. Our analyses of PHMC position were based on within‐subject side‐to‐side comparisons of the PHMC locations, which allowed us to control for differences in knee size between study participants.

Another benefit of our study design is that the loading environment of the knee was controlled by imaging subjects when their knees were in extension (0°) under the same non‐weight‐bearing, unloaded conditions. When the study was designed, we had the capacity to apply compressive loading to the leg through the plantar aspect of each subject's foot. However, we did not have the capability to measure how the intersegmental tibiofemoral loads would be distributed between the medial and lateral compartments of the tibiofemoral joint. This would have produced uncontrolled loading of the menisci. Measurement of compartment‐specific loading would have required us to measure the three‐dimensional anatomic alignment of the entire lower extremity relative to the three‐dimensional orientation of the applied/measured resultant compressive loads and moments acting on the plantar aspect of each subject's foot. Our next step will be to expand on this study by calculating meniscus strains and PHMC positions during compressive loading of the knee with a known and controlled load distribution between the medial and lateral compartments.

This study was further strengthened by using within‐subject comparisons of PHMC position, with the contralateral uninjured knee of ACL‐injured subjects serving as a surrogate for pre‐injury knee geometry. This approach was validated by our prior prognostic ACL injury risk factor research that demonstrated a high degree of side‐to‐side symmetry in tibial and femoral articular surface geometry between the normal knees of control subjects with no history of knee injury or disease [10, 20, 21, 22]. Meniscus segmentations in this prior work were also shown to be highly reliable between multiple MRI acquisitions within the same subjects. Consistent with these findings, the current study demonstrated no significant side‐to‐side differences in PHMC position within control subjects in the sex‐specific (Table 2) or pooled analyses (Table S4). In the data analyzed from the male and female controls as a combined group (sample size of 88 subjects), each of the three components of the PHMC position vectors in the lateral and medial tibial compartments was nearly identical between knees. The observed differences in PHMC position in control subjects were orders of magnitude less than the in‐plane MRI voxel dimensions of 0.3 mm × 0.3 mm (Table S4) and the ACL‐injured to normal knee PHMC position differences (Table 1). Ultimately, these findings support our decision to use the contralateral normal knee of ACL‐injured subjects as a surrogate for PHMC position prior to the index knee injury.

Our prior ACL injury risk factor research [20] conducted in this same cohort further demonstrated no significant side‐to‐side differences in tibial plateau slope/concavity within control subjects with normal knees. Therefore, plateau slope was not included as a covariate in our analyses in the current PHMC study. Given the within‐subject study design and high degree of geometric symmetry between control knees (i.e., articular surface geometry and PHMC position), the primary distinguishing factor between ACL‐injured and contralateral normal knees was the presence/absence of the ACL. Accordingly, the differences in PHMC position observed between ACL‐injured and contralateral normal knees soon after noncontact ACL injury can be associated, at least in part, with ACL deficiency.

It is also important to point out that this study was not powered to make male‐to‐female comparisons of PHMC position, as it was based on additional analyses of data acquired from a prior study performed by our research group that had a central focus of identifying the risk factors for a first‐time noncontact ACL tear [10, 20, 21, 22, 23]. However, many of the differences in PHMC position change between ACL‐injured males and females were statistically significant, indicating that the study was adequately powered for sex‐based comparisons to be made. Additionally, we did not collect data on patient‐reported outcomes, and we were unable to measure patient function because the study participants had ACL‐deficient knees. These considerations will be included in our future work.

One potential limitation of this study is that the out‐of‐plane voxel resolution in our MRI protocol was larger than the in‐plane resolution. However, we do not feel this created partial volume effects that confounded the measurements of PHMC positions in the midportion of the medial and lateral compartments of the tibia. The magnitude of the observed differences in PHMC position should be interpreted in conjunction with the in‐plane resolution (0.3 mm × 0.3 mm), which corresponds to the plane in which the anterior‐posterior and superior‐inferior PHMC positions were measured. Although the out‐of‐plane resolution (1.2 mm) has the potential to introduce confounding due to partial volume effects, our analyses focused on the mid‐medial and mid‐lateral tibial compartments, which do not exhibit appreciable changes in cartilage curvature or thickness in the medial‐lateral direction. In contrast, if this study had included characterization of the tibial spines, which have pronounced medial‐lateral variations in cartilage curvature and thickness, partial volume effects would have required specific consideration. With this in mind, all statistically significant differences in PHMC position between ACL‐injured and contralateral normal knees were substantially greater in magnitude than the in‐plane resolution of the MRI scans. Although smaller voxel dimensions are generally desired, our choice of voxel size reflected a necessary trade‐off between spatial resolution and total scan acquisition time. Our MRI protocol included multiple MRI sequences for both knees that approached the practical limits of what study participants could tolerate without introducing motion artifact. This is always a concern in a study such as ours, which scanned 88 subjects with varying tolerances for remaining motionless in the scanner, requiring us to strike a balance between acquisition time and voxel dimensions.

While the position of each PHMC was determined in three dimensions, complete segmentations of the entire lateral and medial menisci were not performed. This was primarily due to the inability of the T1 FFE MRI acquisition protocol to effectively capture the central meniscal root insertions and the meniscal‐joint capsule interfaces in both compartments. Without complete sagittal segmentations, the three‐dimensional structure of the entire meniscus could not be characterized. Therefore, our study did not determine if ACL injury resulted in additional positional changes to the menisci beyond the mid‐compartment regions of the posterior meniscal horns, in which each PHMC position was determined. This was done to maintain continuity with prior work that analyzed posterior horn meniscus wedge angle as a risk factor for ACL injury and to concentrate our analysis on the region of the lateral and medial menisci that is injured most frequently in combination with ACL trauma [27, 28]. Future studies that fully characterize the menisci are needed to comprehensively assess positional changes in all meniscal regions resulting from ACL injury.

As discussed previously, subjects in this study were not weight‐bearing at the time of MRI acquisition. This is an important consideration because the meniscus deforms in response to loading associated with articulation of the femur relative to the tibia during normal knee function. While the placement of each subject in the MRI scanner and RF coil was completed by the same examiner using the same approach for all study subjects to avoid confounding loading conditions between knees, this environment was not representative of that in which the knee joint functions during daily activity. For example, the unloaded environment of the MRI scanner did not replicate the changes in compressive loads, internal–external rotation, or differences in varus–valgus alignment between ACL‐injured and normal knees that occur during weight‐bearing conditions. Further, prior research indicates that meniscal translation through flexion and extension is greater during weight‐bearing conditions than during non‐weight‐bearing conditions [29]. Unlike the rigid bony structures of the tibia and femur, the menisci have viscoelastic material properties and deform with a temporal response to loading, which could affect meniscal position. When a compressive load is applied across the tibiofemoral joint, the menisci displace radially and circumferentially to distribute and transfer the intersegmental compressive and shear forces between the tibia and femur [30, 31]. Therefore, during activity, it is likely that the magnitude of observed posterior‐directed PHMC position change in ACL‐injured knees would be altered due to the forces experienced by the menisci during weight‐bearing conditions. Future research should consider studying the position and morphometry of the meniscus during weight‐bearing conditions that replicate the loads associated with daily activity.

Based on the results of this study, it is important for future studies of PTOA to consider including meniscus position as a covariate in statistical analysis, in addition to the position of the tibia relative to the femur. This approach may provide insight into the mechanism that produces changes in both articular cartilage thickness and the underlying matrix components following ACL injury. Finally, the substantial and highly variable position of the menisci relative to the tibia in ACL‐injured knees soon after the index trauma suggests that the boundaries of the menisci should not be used as landmarks to establish ROIs in articular cartilage. An alternative approach would be to use bone‐referenced ROIs to eliminate the effects of the variable meniscus position changes produced by injury and disease.

## Author Contributions

B.T.H. participated in data analysis, interpretation of the statistical analysis, construction of figures and tables, wrote the manuscript as the primary author of the study, and approved the submitted and final versions. D.S. made substantial contributions to research design and the acquisition, analysis, and interpretation of data. Approved the submitted and final versions. E.C.A. made substantial contributions to research design and the acquisition, analysis, and interpretation of data. Approved the submitted and final versions. N.F. participated in data interpretation, preparation of the manuscript, and approval of the submitted and final versions. T.W.T. participated in establishing the MRI sequences and protocol, data interpretation, preparation of the manuscript, and approval of the submitted and final versions. M.F. participated in data interpretation, preparation of the manuscript, and approval of the submitted and final versions. M.G.‐M. participated in data interpretation, preparation of the manuscript, and approval of the submitted and final versions. P.M.V. participated in data analysis, interpretation, and preparation of the manuscript, and approval of the submitted and final versions. B.D.B. is responsible for the development of the study hypothesis, design of the investigation, and development of the study protocol. Acquired funding for the investigation and obtained approval for the study from the IRB. Participated in data acquisition, interpretation of the statistical analysis, worked with the first author to write and review the manuscript, and approved the submitted and final versions.

## Conflicts of Interest

The authors declare no conflicts of interest.

## Supporting information

Supporting File
