# Supplementary material for: Posterior Horn Meniscus Centroid Position Is Altered Soon After Noncontact ACL Injury in Males and Females
Source: J Orthop Res. 2026 Apr 27;44(5):e70208. doi: 10.1002/jor.70208 (PMC13112327; doi:10.1002/jor.70208)
Supplement: Supplementary file 1 — Supporting File [file JOR-44-0-s001.docx]

**Supplemental Text For**

**Posterior horn meniscus centroid position is altered soon after non-contact ACL injury in males and females**

**Benjamin T. Hamilton^1^, Daniel Sturnick^2^, Erin C. Argentieri^3^, Niccolo Fiorentino^1,4^, Timothy W. Tourville^1,5^, Matthew Failla^1,5^, Mack Gardner-Morse^1^, Pamela M. Vacek^6^,**

**and Bruce D. Beynnon^1,4^**

^1^Department of Orthopaedics and Rehabilitation, Robert Larner M.D. College of Medicine, University of Vermont, Burlington, Vermont, USA

­^2^Stryker Spine, Stryker, Leesburg, VA, USA

^3^Department of Radiology and Imaging, Hospital for Special Surgery, New York, NY, USA

^4^Department of Mechanical Engineering, University of Vermont, Burlington, VT, USA

^5^Department of Rehabilitation and Movement Science, University of Vermont, Burlington, VT, USA

^6^Biomedical Statistics Research Core, Robert Larner M.D. College of Medicine, University of Vermont, Burlington, Vermont, USA

**Corresponding Author:**

Bruce D. Beynnon, Ph.D.

McClure Professor of Musculoskeletal Research

McClure Musculoskeletal Research Center

Department of Orthopaedics and Rehabilitation

University of Vermont, Robert Larner M.D. College of Medicine

Burlington, Vermont 05405-0084, USA.

Phone: (802)-656-4257

Fax: (802)-656-4247

Email: [bruce.beynnon@med.uvm.edu](mailto:bruce.beynnon@uvm.edu)

**Supplementary Material**

**Supplement 1: Statistical Methods for Between-Sex and Between-Knee Compartment Comparisons of PHMC Position**

Unpaired two-tailed T-tests of the ACL-injured to contralateral normal knee differences in PHMC position were performed between the male and female subject groups in the AP, ML, and SI directions for the lateral and medial compartments to determine if there was a sex effect associated with PHMC position. Equal variances were assumed and an alpha value of 0.050 was used. In addition, paired two-tailed T-tests were used to determine if there were significant differences in PHMC position between the lateral and medial compartments. P-values were calculated for the male and female groups in the AP, ML, and SI directions using an alpha value of 0.050.

**Supplement 2: Statistical Methods for Analyses of the Position of the Tibia Relative to the Femur**

Additional analyses of AP-directed differences in the position of the tibia relative to the femur between ACL-deficient and contralateral normal knees were completed for this same cohort. Unlike the primary comparisons of differences in PHMC position relative to the tibia, the tibial position differences were determined by comparing the location of the origin of the tibial coordinate system relative to the origin of the described bone-fixed femoral coordinate system (Figure S-3). In this coordinate system, the x-axis was defined as the anterior-posterior (AP) directed axis, the y-axis defined as the medial-lateral (ML) directed axis, and the vertical z-axis defined as the superior-inferior (SI) directed axis. Positive differences in tibiofemoral position indicated anterior-, lateral-, and superior-directed differences between knees. Negative differences in tibiofemoral position represent posterior-, medial-, and inferior-directed differences between knees.

Within-subject side-to-side differences in the position of the tibia relative to the femur position were calculated between ACL-injured and contralateral normal knees. In ACL-injured subjects, differences in tibiofemoral position were calculated by subtracting the position of the tibia relative to the femur of each contralateral normal knee from the position of the tibia relative to the femur of their corresponding ACL-injured knee in the AP, ML, and SI directions. Paired two-tailed T-tests using a significance level of 0.050 were performed in the lateral and medial compartments for the male and female groups to determine if there are significant differences in tibiofemoral position between ACL-injured and normal knees with no history of injury or disease.

Differences in the position of the tibia relative to the femur were also calculated between normal knees within control subjects. The ACL-injured knees of each case subject were matched by side to the corresponding normal knees of each control subject. Side-to-side differences in tibiofemoral position were then calculated using the same order of subtraction as was used within ACL-injured subjects in the AP, ML, and SI directions (Figure S-1). Paired two-tailed T-tests using a significance level of 0.050 were completed for the male and female groups to determine if there are significant differences in tibiofemoral position between the normal knees of individuals with healthy knees.

**Figure S-1:** Chart showing the PHMC position difference comparisons made between ACL-injured and contralateral normal knees of case subjects and between both normal knees of matched control subjects. In control subjects, each normal knee was matched by side corresponding to the sidedness of the ACL injury to enable consistency in the difference calculations. PHMC position differences between normal control subject knees were calculated by subtracting the position of Normal Knee 2 (matched by side to contralateral normal knee of the case subject) from Normal Knee 1 (matched by side to the ACL-injured knee of the case subject) in the AP, ML, and SI directions. For example, if a case subject had an ACL-injury to his or her left knee, the PHMC position of the right knee of the matched control subject (Normal Knee 2) would be subtracted from the PHMC position of the control subject’s left knee (Normal Knee 1). This process would be completed in all three anatomical directions. Within ACL-injured subjects, PHMC position differences were calculated by subtracting the PHMC positions of each contralateral normal knee from the PHMC positions of their corresponding ACL-injured knee in all three anatomical directions.


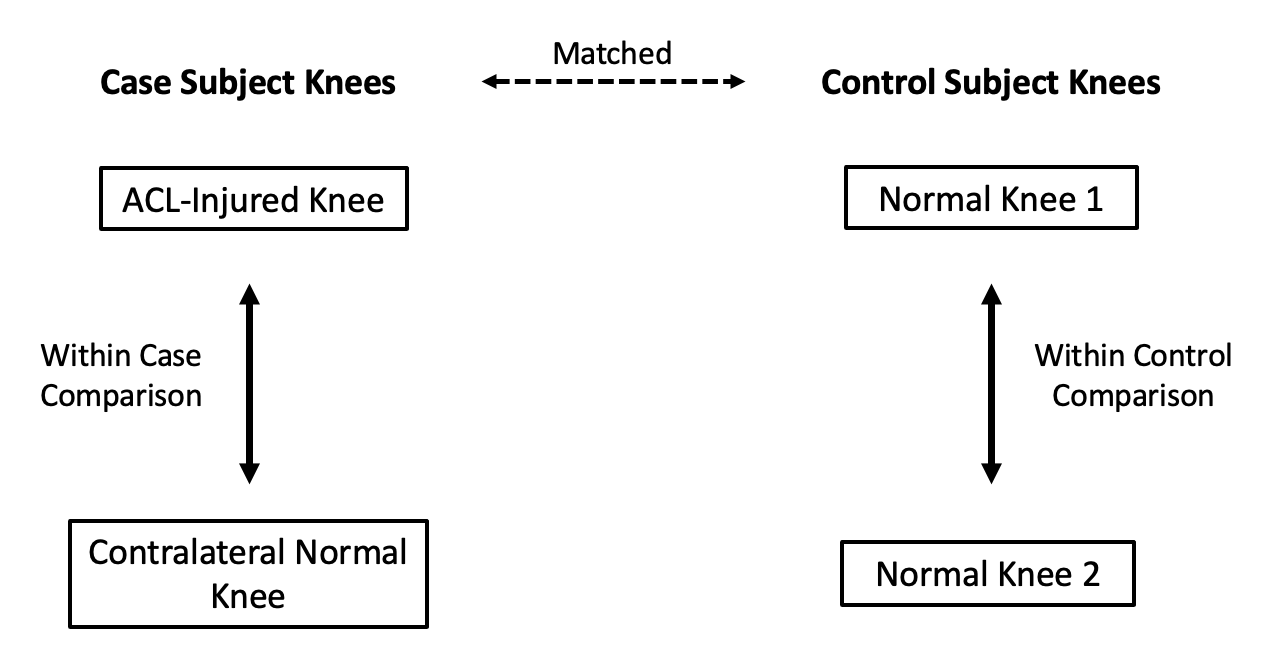


**Table S-1:** Mean differences in the position of the tibia relative to the femur, tibiofemoral internal-external rotation, and knee flexion-extension between ACL-injured and contralateral normal knees within case subjects. Positive mean differences in tibial position indicate anterior-, lateral-, and superior-directed differences in the position of the tibia relative to the femur between ACL-injured and contralateral normal knees. Negative mean differences indicate posterior-, medial-, and inferior-directed differences in tibial position within case subjects. Rotation is positive for internal rotation of the tibia relative to the femur and negative for external tibial rotation relative to the femur. Flexion is positive when the tibia is flexed relative to the femur and negative when hyperextended relative to the femur. All position values are displayed in either millimeters (mm) or degrees and were calculated in alignment with the scheme outlined in Figure S-1.

|  |  | **ACL-Injured Subject Side-to-Side Differences** | | | | |
| --- | --- | --- | --- | --- | --- | --- |
|  |  | **AP (X)**  (mm) | **ML (Y)**  (mm) | **SI (Z)**  (mm) | **Internal-External Rotation**  (deg) | **Flexion-Extension**  (deg) |
| **Male** | Mean (95% CI) | 4.28 ± 0.82 | -0.40 ± 0.47 | 0.76 ± 0.29 | 0.08 ± 1.00 | 0.15 ± 0.53 |
|  | Max | -1.7 | 3.8 | 4.9 | 11.3 | 5.3 |
|  | Min | 19.8 | -6.3 | -2.1 | -7.8 | -5.2 |
|  | SD | 3.94 | 2.24 | 1.40 | 4.80 | 2.55 |
|  | P-Value | <0.001 | 0.365 | 0.009 | 0.932 | 0.759 |
|  | | | | | | |
| **Female** | Mean (95% CI) | 2.35 ± 0.54 | -0.45 ± 0.40 | 0.42 ± 0.24 | -0.56 ± 0.98 | 0.58 ± 0.46 |
|  | Max | -2.1 | 3.3 | 3.0 | 9.2 | 8.0 |
|  | Min | 10.8 | -6.9 | -2.3 | -9.9 | -3.8 |
|  | SD | 2.59 | 1.90 | 1.15 | 4.67 | 2.21 |
|  | P-Value | <0.001 | 0.069 | 0.006 | 0.355 | 0.046 |
|  | | | | | | |
| **Combined Male and Female** | Mean (95% CI) | 2.94 ± 0.66 | -0.43 ± 0.42 | 0.52 ± 0.26 | -0.36 ± 0.98 | 0.45 ± 0.48 |
|  | Max | -2.1 | 3.8 | 4.9 | 11.3 | 8.0 |
|  | Min | 19.8 | -6.9 | -2.3 | -9.9 | -5.2 |
|  | SD | 3.17 | 2.00 | 1.23 | 4.69 | 2.31 |
|  | P-Value | <0.001 | 0.045 | <0.001 | 0.471 | 0.074 |

**Table S-2**: Mean side-to-side differences in the position of the tibia relative to the femur, tibiofemoral internal-external rotation, and knee flexion-extension between normal control knees within control subjects. Positive mean differences in tibial position indicate anterior-, lateral-, and superior-directed differences in the position of the tibia relative to the femur between the normal control knees. Negative mean differences indicate posterior-, medial-, and inferior-directed differences in tibial position between normal control knees. Rotation is positive for internal rotation of the tibia relative to the femur and negative for external tibial rotation relative to the femur. Flexion is positive when the tibia is flexed relative to the femur and negative when hyperextended relative to the femur. All position values are displayed in either millimeters (mm) or degrees and were calculated in alignment with the scheme outlined in Figure S-1.

|  |  | **Control Subject Side-to-Side Differences** | | | | |
| --- | --- | --- | --- | --- | --- | --- |
|  |  | **AP (X)**  (mm) | **ML (Y)**  (mm) | **SI (Z)**  (mm) | **Internal-External Rotation** (deg) | **Flexion-Extension**  (deg) |
| **Male** | Mean (95% CI) | 0.05 ± 0.40 | 0.06 ± 0.36 | 0.27 ± 0.26 | -1.83 ± 0.81 | -0.09 ± 0.39 |
|  | Max | -4.5 | 2.9 | 2.8 | 5.1 | 3.8 |
|  | Min | 3.6 | -3.2 | -1.9 | -9.9 | -4.5 |
|  | SD | 1.93 | 1.71 | 1.24 | 3.89 | 1.88 |
|  | P-Value | 0.895 | 0.848 | 0.269 | 0.021 | 0.803 |
|  | | | | | | |
| **Female** | Mean (95% CI) | 0.45 ± 0.33 | -0.34 ± 0.39 | -0.08 ± 0.23 | 0.37 ± 0.92 | 0.51 ± 0.45 |
|  | Max | -2.9 | 3.9 | 2.7 | 8.4 | 6.6 |
|  | Min | 5.5 | -4.9 | -3.9 | -10.1 | -3.3 |
|  | SD | 1.60 | 1.86 | 1.11 | 4.40 | 2.15 |
|  | P-Value | 0.031 | 0.159 | 0.567 | 0.512 | 0.067 |
|  | | | | | | |
| **Combined Male and Female** | Mean (95% CI) | 0.33 ± 0.36 | -0.22 ± 0.38 | 0.03 ± 0.24 | -0.31 ± 0.91 | 0.33 ± 0.43 |
|  | Max | -4.5 | 3.9 | 2.8 | 8.4 | 6.6 |
|  | Min | 5.5 | -4.9 | -3.9 | -10.1 | -4.5 |
|  | SD | 1.71 | 1.82 | 1.15 | 4.35 | 2.08 |
|  | P-Value | 0.073 | 0.268 | 0.833 | 0.512 | 0.142 |

**Table S-3:** Mean differences in posterior horn meniscus centroid (PHMC) position between ACL-injured and contralateral normal knees within case subjects for males and females as a combined group. Positive mean differences indicate anterior-, lateral-, and superior-directed differences in PHMC position between ACL-injured and contralateral normal knees within case subjects. Negative mean differences represent posterior-, medial-, and inferior-directed differences in PHMC position between ACL-injured and contralateral normal knees. All values are displayed in millimeters (mm) and were calculated by subtracting the PHMC position of the contralateral normal knee from PHMC position of the ACL-injured knee within case subjects.

|  |  | **ACL-Injured Subject Side-to-Side Differences** | | | | | |
| --- | --- | --- | --- | --- | --- | --- | --- |
|  |  | **Lateral Compartment** | | | **Medial Compartment** | | |
|  |  | **AP (X)**  (mm) | **ML (Y)**  (mm) | **SI (Z)**  (mm) | **AP (X)**  (mm) | **ML (Y)**  (mm) | **SI (Z)**  (mm) |
| **Combined Male and Female** | Mean PHMC Position  (95% CI) | -0.99 ± 0.42 | 0.50 ± 0.75 | -0.14 ± 0.28 | -1.16 ± 0.48 | -0.11 ± 0.44 | -0.06 ± 0.22 |
|  | Max | 3.3 | 7.0 | 4.4 | 4.5 | 5.0 | 2.3 |
|  | Min | -7.6 | -8.0 | -5.0 | -7.2 | -5.0 | -3.6 |
|  | SD | 1.99 | 3.59 | 1.35 | 2.31 | 2.11 | 1.07 |
|  | P-Value | <0.001 | 0.195 | 0.318 | <0.001 | 0.615 | 0.572 |

**Table S-4:** Mean side-to-side differences in posterior horn meniscus centroid (PHMC) position between normal control knees for males and females as a combined group. Positive mean differences indicate anterior-, lateral-, and superior-directed differences in PHMC position between the normal knees within control subjects. Negative mean differences represent posterior-, medial-, and inferior-directed differences in PHMC position between normal knees within control subjects. All values are displayed in millimeters (mm) and were calculated using the matching scheme described in Figure S-1.

|  |  | **Control Subject Side-to-Side Differences** | | | | | |
| --- | --- | --- | --- | --- | --- | --- | --- |
|  |  | **Lateral Compartment** | | | **Medial Compartment** | | |
|  |  | **AP (X)**  (mm) | **ML (Y)**  (mm) | **SI (Z)**  (mm) | **AP (X)**  (mm) | **ML (Y)**  (mm) | **SI (Z)**  (mm) |
| **Combined Male and Female** | Mean PHMC Position  (95% CI) | 0.06 ± 0.34 | 0.20 ± 0.62 | 0.08 ± 0.27 | -0.27 ± 0.40 | 0.16 ± 0.49 | 0.09 ± 0.25 |
|  | Max | 4.0 | 7.0 | 2.8 | 5.5 | 6.0 | 3.1 |
|  | Min | -4.2 | -8.0 | -3.6 | -4.3 | -5.0 | -2.9 |
|  | SD | 1.61 | 2.94 | 1.30 | 1.93 | 2.36 | 1.18 |
|  | P-Value | 0.737 | 0.516 | 0.577 | 0.199 | 0.529 | 0.474 |

**Figure S-2:** Femoral coordinate system used for analysis of the position of the tibia relative to the femur described by Pius et al.^1^ The position of the tibia relative to the femur was measured in a bone-based coordinate system in the femur using the approach described by Kauffmann et al.^2^ A cylinder (shown in grey) was fit to the three-dimensional manually digitized sagittal plane segmentations of the lateral and medial femoral condyle subchondral bone surfaces using a nonlinear least squares approach (MATLAB function lsqnonlin, Mathworks, Natick, MA USA).^2, 3^ The medial-lateral directed axis of the femoral coordinate system was defined as the longitudinal axis of the cylinder. A center point was then identified in an axial cross-section of the femoral shaft where the trochlear groove and femoral condyles intersect at the center of the femoral notch. The point along the medial-lateral axis of the cylinder closest and perpendicular to this center point was defined as the origin of the femoral coordinate system axes. The superior-inferior direction was defined as a line passing through the centroid of the transverse plane of the outer cortex of the femoral shaft and through the origin of the femoral coordinate system. The superior-inferior axis was defined as the cross product of the anterior-posterior and medial-lateral axes. The anterior-posterior directed axis was defined as the cross product of the medial-lateral and superior-inferior axes. The transverse plane was located three times the femoral shaft width distal to the origin of the coordinate system. The anterior, lateral, and superior directions were defined by positive values, and the posterior, medial, and inferior directions were represented by negative values. Right-leg femurs were reflected in the medial-lateral direction to match left-leg femurs. The reliability of establishing the femoral coordinate system in this manner has been described.^4^

**
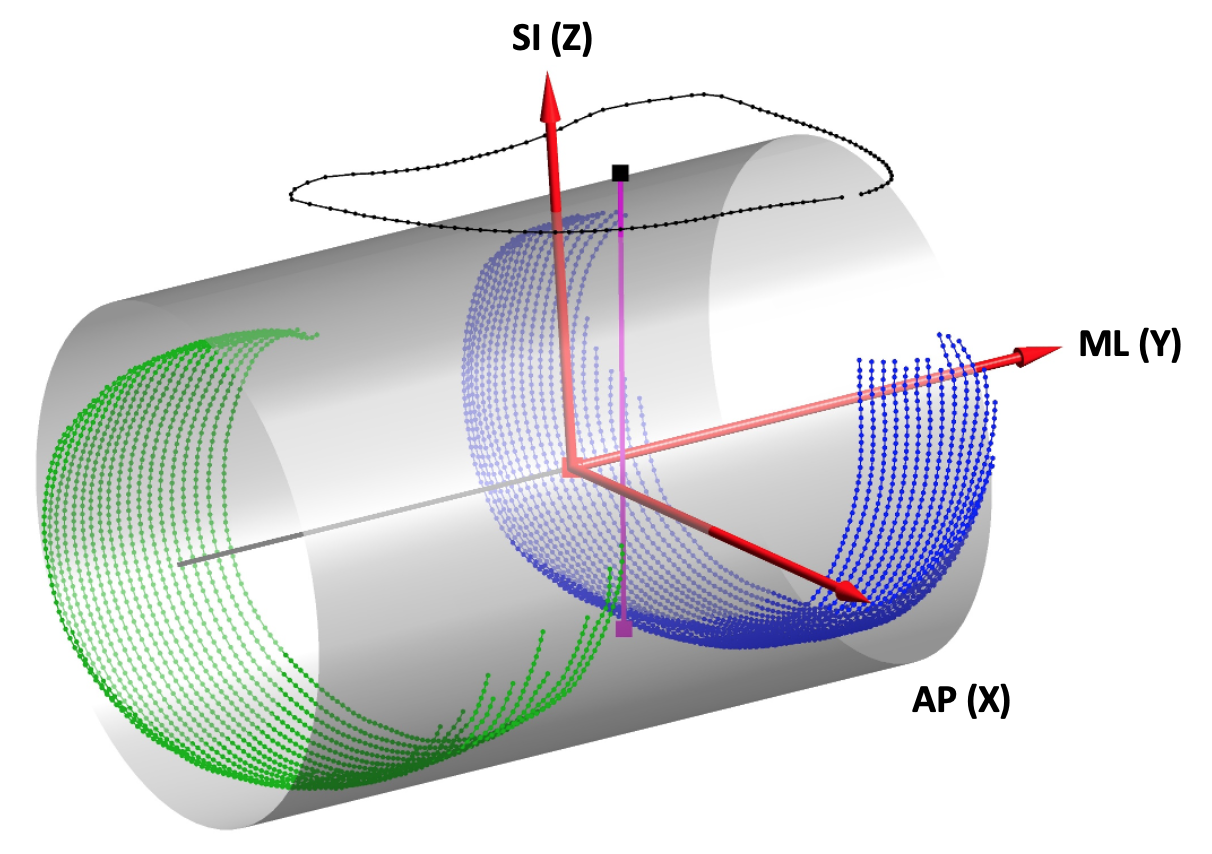
**

**Figure S-3:** Femoral coordinate system used for analysis of the position of the tibia relative to the femur shown with the tibial coordinate system used to determine PHMC position relative to the tibia.

**
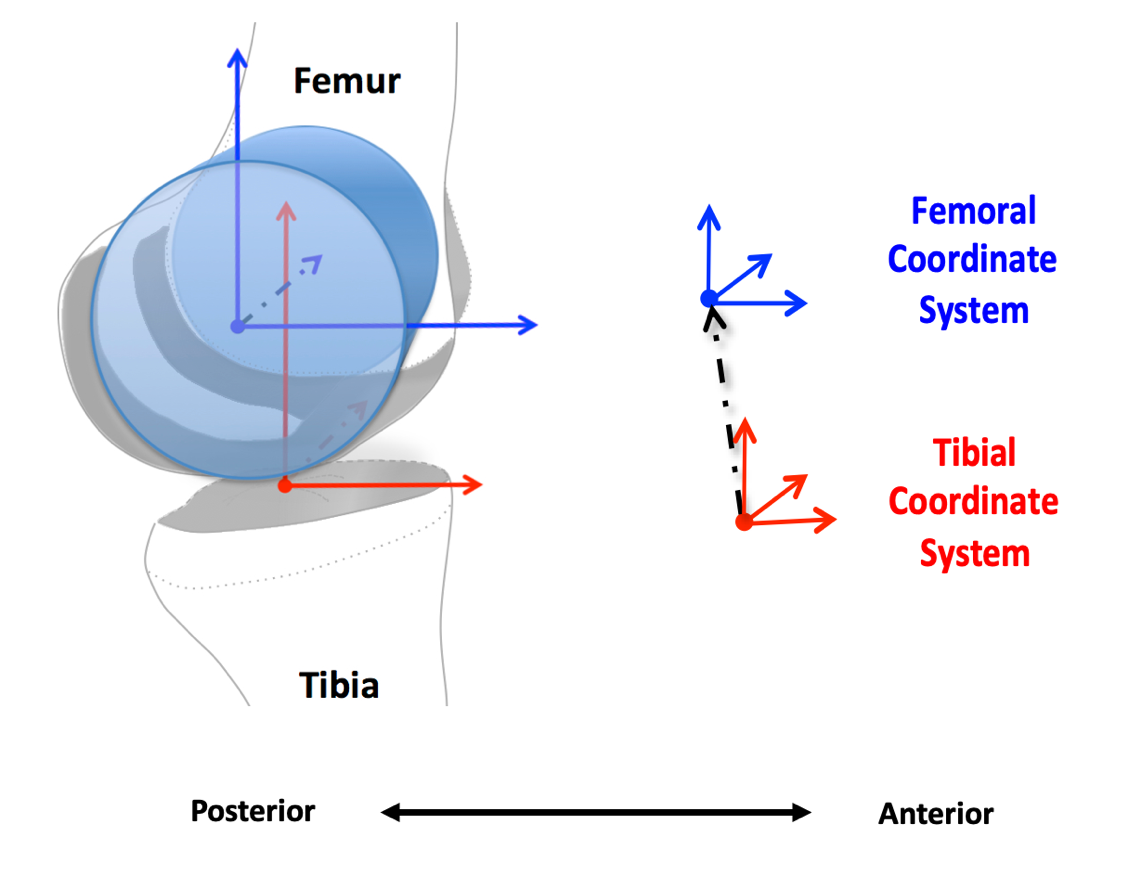
**

**References**

1. Pius AK, Beynnon BD, Fiorentino N, et al. Articular cartilage thickness changes differ between males and females 4 years following anterior cruciate ligament reconstruction. *J Orthop Res*. 2022;40(1):65-73.

2. Kauffmann C, Gravel P, Godbout B, et al. Computer-aided method for quantification of cartilage thickness and volume changes using MRI: validation study using a synthetic model. *IEEE Trans Biomed Eng*. 2003;50(8):978-988.

3. Favre J, Erhart-Hledik JC, Chehab EF, Andriacchi TP. Baseline ambulatory knee kinematics are associated with changes in cartilage thickness in osteoarthritic patients over 5 years. *J Biomech*. 2016;49(9):1859-1864.

4. Raynauld JP, Kauffmann C, Beaudoin G, et al. Reliability of a quantification imaging system using magnetic resonance images to measure cartilage thickness and volume in human normal and osteoarthritic knees. *Osteoarthritis Cartilage.* 2003;11(5):351-360.
